# A General Model for Biofilm-Driven Microbial Electrosynthesis of Carboxylates From CO_2_

**DOI:** 10.3389/fmicb.2021.669218

**Published:** 2021-06-04

**Authors:** Oriol Cabau-Peinado, Adrie J. J. Straathof, Ludovic Jourdin

**Affiliations:** Department of Biotechnology, Faculty of Applied Sciences, Delft University of Technology, Delft, Netherlands

**Keywords:** microbial electrosynthesis, bioelectrochemical system, microbial kinetics, mathematical model, CO_2_ reduction, chain elongation

## Abstract

Up to now, computational modeling of microbial electrosynthesis (MES) has been underexplored, but is necessary to achieve breakthrough understanding of the process-limiting steps. Here, a general framework for modeling microbial kinetics in a MES reactor is presented. A thermodynamic approach is used to link microbial metabolism to the electrochemical reduction of an intracellular mediator, allowing to predict cellular growth and current consumption. The model accounts for CO_2_ reduction to acetate, and further elongation to n-butyrate and n-caproate. Simulation results were compared with experimental data obtained from different sources and proved the model is able to successfully describe microbial kinetics (growth, chain elongation, and product inhibition) and reactor performance (current density, organics titer). The capacity of the model to simulate different system configurations is also shown. Model results suggest CO_2_ dissolved concentration might be limiting existing MES systems, and highlight the importance of the delivery method utilized to supply it. Simulation results also indicate that for biofilm-driven reactors, continuous mode significantly enhances microbial growth and might allow denser biofilms to be formed and higher current densities to be achieved.

## Introduction

Microbial electrosynthesis (MES) is based on the use of microorganisms that can reduce CO_2_ to industrially relevant products (i.e., alcohols, carboxylic acids) by using electrons coming from a solid-state electrode (Lovley and Nevin, 2013; Jourdin and Strik, 2017; Kerzenmacher, 2017). MES is a promising technology to satisfy the growing demand for commodity and specialty chemicals, and has the potential to increase the value of the electrical energy produced from renewable sources (Lewis and Nocera, 2006). Until now, research on MES has been primarily focused on developing the technology by means of studying its fundamentals (e.g., electron transfer mechanisms, metabolic routes used for reducing CO_2_) and improving the efficiency of crucial components (e.g., microorganisms, cathode structure, and material) (Jourdin and Burdyny, 2020; Prévoteau et al., 2020). Even though significant progress has been achieved on these aspects, MES technology still needs to be pushed to higher performance to reach industrial viability (Jourdin et al., 2020). In that sense, rate-limiting steps, scalability, and system optimization are key aspects that need to be assessed. Initial work in all those directions has been published in the last decade (Giddings et al., 2015; Alqahtani et al., 2018; Enzmann et al., 2019; Rosa et al., 2019), but progress has been modest.

MES is a complex system that combines both electrochemistry and biotechnology. When trying to improve reactor performance, all physical, chemical, and biological processes occurring simultaneously have to be properly regulated. A major breakthrough would require a deeper understanding of this inherent complexity. To that end, computational models are a systematic approach that can be used for testing hypotheses and obtain knowledge on the described system, as pointed out by Korth and Harnisch (2017) in their detailed review on modeling microbial electrosynthesis.

When looking at the biocatalyst itself, metabolic modeling allows for an in-depth analysis of the molecular and biochemical mechanisms occurring within a particular microorganism. These complex mathematical expressions encompass all major metabolic pathways, and simulate them in perspective of the entire metabolic network. Pandit and Mahadevan (2011) used flux balance analysis to develop one of the first computational genome-scale metabolic models, and used it to characterize the role of bioelectrosynthesis in chemical production. Their model was based on the genome of *Escherichia coli*, and showed that trade-offs between improving growth rates and yields could exist. Kracke and Krömer (2014) used elementary mode analysis to create multiple core networks of metabolic carbon pathways, and found that the yield obtained with electrical enhancement depends strongly on the electron transport mechanism. Marshall et al. (2017) constructed three full genome-scale metabolic models that in combination with flux balance analysis, allowed them to predict the metabolic activity of different microbial communities. Their results identified the main metabolic pathways in those systems, as well as demonstrating the possibility of multiple species being active within a very limited space near an electrode. Metabolic network models are of great use when exploring suitable MES processes, as well as when studying the pathways present within a particular microorganism. However, these are complex mathematical expressions that require prior knowledge on the genome and transcriptome of the studied organism. Moreover, data on interactions between species in mixed microbial culture biofilms, biofilm structure, and mass transport phenomena are needed when extrapolating results to a biofilm superstructure. Since this information is largely unknown in MES to date, and metabolic models are mostly focused on microbial cells and their immediate surroundings, these models are hardly suited for a more generalized study at a reactor scale.

To date, few modeling studies have been published on microbial electrosynthesis and the dynamics between microorganisms and operating conditions. Kazemi et al. (2015) modeled a biofilm-based MES cathode with a pure culture producing acetate using a conductive biofilm approach. The model allowed them to study current density and biofilm thickness on different CO_2_ concentrations and applied cathodic potentials. Their model showed that high CO_2_ concentrations decreased coulombic efficiency, while a higher cathodic potential increased the coulombic efficiency. Gadkari et al. (2019) performed a study of the interdependence of some operating parameters in a MES system using a bioanode. They developed a two-chamber model with two cell populations, allowing them to analyze the effect of parameters such as initial substrate concentration and operation cycle time on MES performance. Their results showed that reducing the operation cycle time favored production rates, but decreased substrate utilization and coulombic efficiency. Abel and Clark (2020) very recently modeled a biomass-producing system that reduces CO_2_ into formate electrochemically, which is then used aerobically by planktonic cells to grow. They were able to study the dynamics between CO_2_, O_2_, and biomass growth as well as the influence of some operational parameters on the general performance of the reactor. O_2_ and CO_2_ mass transfer were found to be limiting the formate-mediated reactor. Their study also indicates that gas recycling to increase overall CO_2_ utilization will be necessary when scaling-up these systems. Salimijazi et al. (2020) also very recently developed a mathematical model to determine the maximum theoretical efficiency of MES processes from electrical power to biofuels. They predicted that by using highly engineered microorganisms, the conversion efficiency to biofuels could increase up to 52%. Their study also shows an interdependence between said efficiency, and biofilm thickness and resistivity. To maintain a given efficiency, if a biofilm resistivity increases its thickness must decrease, while increasing its area.

The use of computational modeling of MES at reactor scale for process understanding and system optimization has clearly been underexplored. Previous modeling papers mainly focused on studying the effects of operational parameters on the general performance of specific MES processes. Moreover, one of the main knowledge gaps in MES is that microbial growth rates and microbial kinetics have not been experimentally elucidated and are thus unknown to date. To achieve a higher process performance, a deeper understanding on the microbial metabolism and production kinetics is necessary. In addition, the study of how microorganisms adapt to changing operational parameters and to different reactor environments (i.e., changing substrate and/or product concentrations) is of crucial importance when elucidating what is limiting MES performance. A general black-box mathematical model allowing for the dynamic description of attached microbial cells, and their interactions with the cathode can help to study such complex environments and potentially elucidate current process bottlenecks. To this end, the objective of this work was to develop a reactor-scale mathematical modeling framework for the study of biofilm-driven microbial electrosynthesis processes with multiple product spectrum and different operational conditions, i.e., batch or continuous mode, continuous or discontinuous CO_2_ supply. To achieve this, a dynamic black-box model of a MES reactor for the reduction of CO_2_ including microbial kinetics with product inhibition and integrated chain elongation, was implemented and solved with the MATLAB software package (MATLAB 2019b).

Since microbial kinetics in MES are not yet available, the biofilm-driven reactor from Jourdin et al. (2019) is used to fit the model and estimate the unknown kinetic parameters. The model is then applied to and validated with experimental data obtained from other studies. First, the capacity of the model to successfully predict different operational conditions is shown by simulating the system from Jourdin et al. (2018). In this first simulation, the same reactor but operated under different dilution rates and feeding strategies is evaluated. Afterward, since not all reported MES reactors reach chain elongation and more than 75% of all MES studies have reported only acetate production (Flexer and Jourdin, 2020), the ability of the model to simulate different product spectrum is also shown. For this purpose, the experimental data from the batch reactor used in Marshall et al. (2013) is used for validation.

## Model Description

### System Overview

**FIGURE 1:**
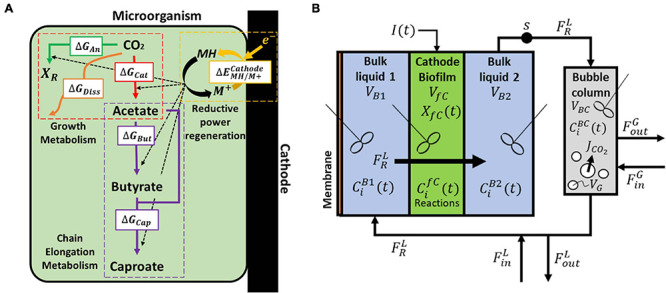
**(A)** Biological and electrochemical reactions occurring within a single cell. The energy generated during the reduction of carbon dioxide (catabolism) is used for biomass formation (anabolism), butyrate and caproate formation (chain elongation), and partly dissipated as heat. The reductive power is regenerated in the bioelectrochemical reduction of the oxidized mediator M^+^ and the excess of reduced mediator is used in the chain elongation metabolism. **(B)** Model domains of the continuous reactor based on Jourdin et al. (2019). CO_2_ is supplied in the external bubble column during liquid recirculation and the catholyte is forced through a porous biocathode.

The model consists of a bioelectrochemical reactor with multiple domains encompassing all subsequent mass balance equations, as well as all electrochemical and biological kinetic reactions. The four domains of the system modeled to simulate reactors from Jourdin et al. are shown in Figure 1B, namely the gas/liquid mass transfer compartment, the cathode biofilm, and both bulk liquid compartments on either side of the cathode/biofilm. The two domains of the modeled reactor from Marshall et al. can be found in Supplementary Figure 1 in the Supplementary Information 1.1. All symbols used and their respective units can be found in the main text in Table 1 and in the Supplementary Information 1.2. Assumptions for all cases are:

**TABLE 1:** Input parameters used in the fitting of the model with their symbols, values, and units.

**Parameter**	**Symbol**	**Case value**	**Units**	**Source**
**Thermodynamics and microbial kinetics**				
Standard Gibbs energy of dissipation for CO_2_	$△G_{Diss}^{CO_{2}}$	1076	kJ/mol_*x*_	[a]
Standard Gibbs energy of M^+^/MH	△G(M/+MH)0	−21.85	kJ/mol_(M*/MH)_	Adapted from [b]
Half-saturation constant for CO_2_	*K*_*CO_2*_	3.8	mol/m^3^	[c]
Half-saturation constant for NH_4_^+^	*K*_*NH_4*_	0.05	mol/m^3^	[d]
Half-saturation constant for MH	*K*_*MH*_	0.1	mol/m^3^	[b]
Half-saturation constant for Acetate	*K*_*Ac*_	0.27	mol/m^3^	[e]
Half-saturation constant for Butyrate	*K*_*But*_	0.076	mol/m^3^	[f]
Critical concentration for Acetate	$C_{\text{Ac}}^{\text{*}}$	800	mol/m^3^	[g]
Critical concentration for Butyrate	$C_{\text{But}}^{\text{*}}$	285	mol/m^3^	[h]
Critical concentration for Caproate	$C_{\text{Cap}}^{\text{*}}$	170	mol/m^3^	[i]
**Electrochemical kinetics**				
Standard heterogeneous electron transfer rate	$k_{e}^{0}$	0.03	1/s	[j]
Transfer coefficient	α	0.5	–	[k]
Standard redox potential of M^+^/MH	*E_M_*	−0.32	V (SHE)	[l]
Cathode potential	*E_C_*	−1.2	V(SHE)	[m]
Number of electrons transferred	n	2	mol_*e–*_/mol_(M*/MH)_	From Eq. 1
**Bulk liquid**				
Bulk liquid volume	$V_{T}^{C}$	370	mL	[m]
Bubble column volume	*V*_BC_	280.9	mL	[m]
Dilution rate	$D_{R}^{L}$	Variable	1/d	[m]
H^+^ concentration	$C_{H^{+}}^{\text{B}}$	10^–5.8^	mol/L	[m]
Gas-liquid mass transfer coefficient	*k_La_*	2.5	1/h	Calculated from [m]
**Initial concentrations**				
Carbon dioxide	$C_{CO_{2}}^{0}$	0	mol/m^3^	[m]
Acetate	$C_{\text{Ac}}^{0}$	30	mol/m^3^	[m]
Butyrate	$C_{\text{But}}^{0}$	0	mol/m^3^	[m]
Caproate	$C_{\text{Cap}}^{0}$	0	mol/m^3^	[m]
**Biofilm**				
Biocathode volume	*V*_*fc*_	25.5	mL	Adapted from [m]
Initial biomass concentration	$C_{X}^{0}$	5.2	mol/m^3^	Adapted from [m]
Initial concentration of MH + M^+^	$C_{MH/M^{+}}^{\text{fc}}$	20	mol/m^3^	[b]
**Constants**				
Faraday constant	*F*	96485.34	C/mol	
Universal gas constant	*R*	8.31	J/(mol K)	
Standard temperature	*T_0_*	298	K	
Working temperature	*T*	305	K	[m]

*[a] Heijnen and Van Dijken (1992), [b] Korth et al. (2015), [c] Nielsen et al. (2019), [d] Pérez et al. (2005), [e] Wiesenborn et al. (1988), [f] Ahring and Westermann (1987), [g] Klemps et al. (1987), [h] Zheng and Yu (2005), [i] Roghair et al. (2018), [j] Ly et al. (2013), [k] Hamelers et al. (2011), [l] Dubouchaud et al. (2018), [m] Jourdin et al. (2019).*

• The biofilm is a continuous phase, thus different microbial species and their distribution are neglected.
• All reactions occur in the biofilm only. Reactions occurring in the bulk liquid are neglected, as the prevalence of the biofilm over suspended cells was demonstrated on their investigation.
• Electrical resistances of the catholyte and the biofilm matrix can be neglected.
• Volumes of all compartments are constant.
• All liquid compartments are well mixed.
• The biofilm domain is also well-mixed (no concentration gradients, see section “Model Assumptions Evaluation”).
• Microorganisms accumulate in the biofilm domain and do not flow out (detachment from the biofilm is neglected).
• pH, electrical potential, and temperature are strictly controlled.

Simulations were performed with a set of mass balances including the exchange rate (flow in and out) from the continuous operation, the net rate of reactions in the biofilm, and the gas/liquid transfer of CO_2_ (in detail in Supplementary Information 1.2). The gas/liquid mass transfer from the gas to the liquid phase was modeled with the overall gas/liquid mass transfer coefficient (*k_La_*). It is possible to simulate reactors with different geometries, cathode properties or cell cultures modifying the obtained mass balance equations.

A general scheme of the microbial catalyst, with all the relevant biological and electrochemical reactions is depicted in Figure 1A, and further described in sections “Electrochemical Reactions”–“Biological Reactions” (Figure 1).

### Electrochemical Reactions

#### Electron Transfer From Cathode to an Intracellular Electron Mediator

In microbial electrosynthesis, electrons must be transferred from the surface of the cathode to the intracellular space of the microorganism likely *via* multiple transmembrane redox centers (e.g., cytochromes) (Morgado et al., 2012). This redox protein chain leads to the reduction of an intracellular redox mediator, which is then used by the microorganism in its metabolism (Hamelers et al., 2011). For simplicity purposes and since intermediate processes are not expected to be limiting the electrochemical rate, the electron transfer between the cathode and the microorganism was assumed to occur by the direct reduction of an oxidized mediator species (M^+^):

(1) M++H++2e←ker-M→kefH

The electrochemical rate $r_{M}^{elec}$ ($mol_{M^{+}}m_{fc}^{-3}s^{-1}$) for Eq. 1 is obtained with the Butler-Volmer equation (Eq. 2). This general model has been successful in the modeling of heterogeneous electron transfer between microorganisms and electrode in microbial fuel cell processes (Zeng et al., 2010; Hamelers et al., 2011).

(2) $$r_{M}^{elec}=k_{e}^{f}C_{M^{+}}C_{H^{+}}-k_{e}^{r}C_{MH}$$

The electrochemical rate coefficients, which account for the electrical potential of the electrode and the mediator, $k_{e}^{f}$ (Eq. 3) and $k_{e}^{r}$ (Eq. 4) are:

(3) $$k_{e}^{f}=k_{e}^{0}exp\left[-\alpha \frac{nF}{RT}\left(E_{C}-E_{M}\right)\right]$$

(4) $$k_{e}^{r}=k_{e}^{0}exp[\left(1-\alpha \right)\frac{nF}{RT}\left(E_{C}-E_{M}\right]$$

To date, the exact mechanism for extracellular electron transfer (EET) is largely unknown. A wide range of different mechanisms has been investigated, from direct electron transfer (Nevin et al., 2010, 2011) to mediated processes (Blanchet et al., 2015; Jourdin et al., 2016). The electron transfer mechanism can be highly dependent on the type of system being studied, hence the aforementioned mathematical expressions were chosen as they allow to mimic different EET mechanisms by adjusting the electrode and the mediator potentials.

#### Current Density

The transfer of electrons from cathode to microorganism results in the observed electric current. This current is determined by a balance between the electrochemical reaction rate $r_{M}^{elec}$ (Eq. 2) and the biological conversion rate. In the present model, the current at the cathode is given by the electrochemical reduction of the redox mediator (Eq. 5). Since the electrochemical rate is defined per biofilm volume, a correction to account for the volume of the biocathode (*V*_*fc*_) domain is included.

(5) $$I=nFr_{M}^{elec}V_{fc}$$

### Biological Reactions

#### Microbial Metabolism

Acetate originates from CO_2_, but the pathways for butyrate and caproate production in MES systems are largely unknown. Acetate elongation can occur *via* multiple pathways, including or not carbon dioxide utilization (Raes et al., 2017; Jourdin et al., 2018; Vassilev et al., 2018). In addition, ethanol has been hypothesized to act as electron donor for the elongation of acetate into longer carboxylates (Ganigué et al., 2015; Batlle-Vilanova et al., 2017; Vassilev et al., 2018). Owing to the high complexity of mixed microbial communities, simplifications are needed when trying to model such environments. The present study approaches this simplification by encompassing all major metabolisms from different cells into one hypothetical black box organism. However, the addition of solventogenesis and chain elongation to the general growth metabolism would require prior knowledge of the exact ratios at which acetate, butyrate, caproate, and ethanol are produced (Heijnen and Van Dijken, 1992; Kleerebezem and Van Loosdrecht, 2010). Since these ratios are not known, the metabolism of the modeled organism can be separated into four different steps, i.e., 1) the energy-generating catabolic reaction, 2) the energy-consuming anabolic reaction for biomass production, 3) the chain elongation metabolism, and 4) the electrochemical regeneration of the reductive power (Figure 1A). The use of a thermodynamic approach allows to account only for end products of the metabolism, bypassing intermediates like ethanol. Hence, for modeling purposes and since this information is currently not available for MES processes, a CO_2_-independent acetate and butyrate elongation pathway not linked to growth is hypothesized. The model includes reaction rates for CO_2_, acetate, butyrate, and caproate. Including reaction rates for compounds that have not been detected would lead to additional kinetic parameters, and these would be unidentifiable. The studies used in this work for parameter fitting and model validation did not detect ethanol or propionate, for example, so reaction rates for these compounds are not included here. To model studies that did measure concentrations of these compounds, model extension is needed.

The general growth stoichiometry of the proposed bacteria is then calculated following a thermodynamic state analysis (Kleerebezem and Van Loosdrecht, 2010). From an energetic point of view, acetate is produced from carbon dioxide to generate energy for all the other reactions occurring within the cell (△*G*_Cat_). A part of that energy is used for butyrate (△*G*_But_) and caproate (△*G*_Cap_) production, as well as for biomass growth and cell maintenance reactions (△*G*_An_), whereas the rest is dissipated as heat (△*G*_*Diss*_). The biomass formula is assumed to be CH_1.8_O_0.5_N_0.2_ (Roels, 1983; Battley, 1987). The catabolic microbial reaction for carbon dioxide reduction to acetate (Eq. 6) and the anabolic reaction for growth (Eq. 7) can be written as follows:

(6) $$2CO_{2}+4MH+3H^{+}=CH_{3}COO^{-}+4M^{+}+2H_{2}O$$

$$CO_{2}+2.1MH+0.2NH_{4}^{+}+1.9H^{+}=$$

(7) $$CH_{1.8}O_{0.5}N_{0.2}+2.1M^{+}+1.5H_{2}O$$

As explained previously, ethanol is not included in the elongation metabolism of the proposed bacteria and the reductive power is assumed to directly come from the redox mediator MH. Then, acetate (Eq. 8) and butyrate (Eq. 9) elongation reactions are described as:

(8) $$2CH_{3}COO^{-}+2MH+3H^{+}=C_{3}H_{7}COO^{-}+2M^{+}+2H_{2}O$$

$$CH_{3}COO^{-}+C_{3}H_{7}COO^{-}+2MH+3H^{+}=$$

(9) $$C_{5}H_{11}COO^{-}+2M^{+}+2H_{2}O$$

The Gibbs energies of reaction $△G_{\text{Cat}}^{0}$, $△G_{\text{An}}^{0}$, $△G_{\text{But}}^{0}$ and $△G_{\text{Cap}}^{0}$ are calculated using the values for the energy of formation obtained from Kleerebezem and Van Loosdrecht (2010), and adapted to reactor conditions (Supplementary Information 1.3). In this model, the mediator couple MH/M^+^ is the only redox mediator species that limit the rate of the redox reactions of the modeled microorganism. The Gibbs energy of formation for this pair was estimated from the standard redox potential and adapted to reactor conditions, as described by Korth et al. (2015). In this study, the standard redox potential of NADH/NAD^+^ was chosen (in detail in the Supplementary Information 1.4).

The catabolic rate (λ_Cat_) is a factor representing how many times the catabolic reaction must occur to supply enough energy for the anabolic, elongation, and dissipation reactions. Using a dissipation energy for chemoautotrophic CO_2_ reducing processes of $△G_{Diss}^{CO_{2}}=1076kJmol_{X}^{-1}$ (Heijnen and Van Dijken, 1992), the catabolic rate (Eq. 10) is calculated:

(10) $$\lambda_{\text{Cat}}=\frac{△G_{Diss}^{CO_{2}}+△G_{\text{An}}}{-△G_{\text{Cat}}+Y_{\text{Ac}}^{But}△G_{\text{But}}+Y_{\text{Ac}}^{\text{Cap}}△G_{\text{Cap}}}$$

The thermodynamic yields $Y_{\text{Ac}}^{But}$ and $Y_{\text{Ac}}^{\text{Cap}}$ are calculated as the ratio between the energies of formation of both butyrate and caproate over acetate. The growth stoichiometry ($Y_{i}^{Met}$) is then obtained by combining both catabolic and anabolic reactions (Eq. 6 and Eq. 7) as $\lambda_{Cat}Y_{i}^{Cat}+Y_{i}^{An}$, resulting in the following general metabolic reaction (Eq. 11):

$$-Y_{CO_{2}}^{Met}CO_{2}-Y_{MH}^{Met}MH-Y_{NH_{4}^{+}}^{Met}NH_{4}^{+}-Y_{H^{+}}^{Met}H^{+}+Y_{X}^{Met}$$

(11) $$CH_{1.8}O_{0.5}N_{0.2}+Y_{\text{Ac}}^{Met}C_{2}H_{3}O_{2}^{-}+Y_{M^{+}}^{Met}M^{+}+Y_{H_{2}O}^{Met}H_{2}O$$

#### Microbial Kinetic Equations

A triple hyperbolic uptake equation accounting for both the carbon and nitrogen sources, as well as for the electron donor is used to describe the carbon dioxide specific uptake rate (Monod, 1949; Bader, 1978; Bae and Rittmann, 1996) (Eq. 12; Parameters in Table 1).

(12) $$q_{{\mathrm{CO}}_{2}}=q_{{\mathrm{CO}}_{2}}^{max}\frac{C_{{\mathrm{CO}}_{2}}}{K_{{\mathrm{CO}}_{2}}+C_{{\mathrm{CO}}_{2}}}\frac{C_{NH_{4}^{+}}}{K_{NH_{4}^{+}}+C_{NH_{4}^{+}}}\frac{C_{\text{MH}}}{K_{\text{MH}}+C_{\text{MH}}}$$

The maintenance coefficient on CO_2_ for anaerobic microorganisms, *m*_CO__2_ ($mol_{CO_{2}}mol_{X}^{-1}h^{-1}$) is estimated with a temperature dependent Arrhenius-type equation (Eq. 13) (Tijhuis et al., 1993). The specific biomass growth rate μ (*h*^−1^) can then be described as a function of the carbon dioxide uptake and maintenance rates (Eq. 14).

(13) $$m_{CO_{2}}=\frac{3.3}{△G_{\text{Cat}}}exp\left[\frac{-69.4}{R}\left(\frac{1}{T}-\frac{1}{T_{0}}\right)\right]$$

(14) $$\mu =\frac{q_{{\mathrm{CO}}_{2}}+m_{{\mathrm{CO}}_{2}}}{Y_{{\mathrm{CO}}_{2}}^{Met}}$$

The specific elongation rates for butyrate and caproate production are then described using double and triple hyperbolic uptake equations, respectively. Jourdin et al. (2018) described a threshold concentration of acetate necessary for chain elongation to occur. A follow-up study suggested that also a threshold concentration of butyrate for caproate production might exist (Jourdin et al., 2019). To incorporate these threshold values into the model, the method proposed by Ribes et al. (2004) is applied to the hyperbolic specific uptake rates for both acetate (Eq. 15) and butyrate (Eq. 16).

(15) $$q_{\text{But}}^{elong}=q_{\text{But}}^{max}\frac{C_{\text{Ac}}-C_{\text{Ac}}^{t}w_{\text{Ac}}}{K_{\text{Ac}}+C_{\text{Ac}}-C_{\text{Ac}}^{t}w_{\text{Ac}}}Z_{\text{Ac}}\frac{C_{\text{MH}}}{K_{\text{MH}}+C_{\text{MH}}}$$

$$q_{\text{Cap}}^{elong}=q_{\text{Cap}}^{max}\frac{C_{\text{Ac}}-C_{\text{Ac}}^{t}w_{\text{Ac}}}{K_{\text{Ac}}+C_{\text{Ac}}-C_{\text{Ac}}^{t}w_{\text{Ac}}}$$

(16) $$Z_{\text{Ac}}\frac{C_{\text{But}}-C_{\text{But}}^{t}w_{\text{But}}}{K_{\text{But}}+C_{\text{But}}-C_{\text{But}}^{t}w_{\text{But}}}Z_{\text{But}}\frac{C_{\text{MH}}}{K_{\text{MH}}+C_{\text{MH}}}$$

Where *w_i_* and *Z_i_* are empirical sigmoidal functions used to ensure the rates have a smooth increase when the concentration reaches the threshold value $C_{i}^{t}$ and to avoid negative values if the threshold is yet to be achieved (Eq. 17 and Eq. 18).

(17) $$w_{i}=\frac{1}{1+exp\left[A_{i}\left(C_{i}^{t}-C_{i}\right)\right]}$$

(18) $$Z_{i}=\frac{1}{1+exp\left[A_{i}\left(T_{i}-C_{i}\right)\right]}$$

The additional tuning parameters *A_i_* and *T_i_* incorporated into the substrate uptake expressions have no biological meaning and are $10/C_{i}^{t}$ and $1.1C_{i}^{t}$, respectively. An elaborated discussion on how to determine these terms can be found in the original paper (Ribes et al., 2004).

The overall coupling between substrate uptake, biomass growth, maintenance, and elongation reactions for all the remaining metabolites (excluding CO_2_) is achieved by using a Herbert-Pirt relation (Eq. 19) and the general metabolic stoichiometry (Eq. 11).

(19) $$q_{i}=Y_{i}^{Met}\mu -m_{{\mathrm{CO}}_{2}}\frac{Y_{i}^{\text{Cat}}}{Y_{{\mathrm{CO}}_{2}}^{\text{Cat}}}+Y_{i}^{But}q_{\text{But}}^{elong}+Y_{i}^{\text{Cap}}q_{\text{Cap}}^{elong}$$

Finally, biological rates for all chemical components are *r_i_ = q_i_C_X_* and *r*_*X*_ = μ*C*_*X*_ for biomass.

#### Carboxylic Acids Inhibition

Carboxylic acids (CAs) are known for their toxicity, which can be attributed to their acid form. The acid form is able to diffuse across the cell membrane and deprotonate in the cytoplasm, generating a pH gradient. In order to maintain homeostasis, cells typically have to use membrane-bound ATPases to expel the excess of protons to the outside. As more ATP is redirected to keep this gradient under control, growth and production yields are substantially decreased (Russell, 1992, 2007). Moreover, the longer the carbon chain, the higher the toxicity of the acid, since long CAs are able to damage the structure of the cell membrane (Roghair et al., 2018).

When modeling microorganisms in MES systems, it is important to account for CAs inhibition. However, product inhibition kinetics in MES remains unknown to date, hence a generalized inhibition model is preferred here. In this study, a linear model is adopted (Ghose and Tyagi, 1979). Product inhibition effect is described by the linear term $\left(1-C_{i}/C_{i}^{*}\right)$, where $C_{i}^{*}$ (*mol m*^−3^) refers to the critical concentration at which the whole metabolism is halted due to the toxicity of the produced compound. Acetate, butyrate, and caproate inhibition terms are then added to the carbon dioxide uptake rate Eq. 12 and to the elongation rates Eq. 15 and Eq. 16 as follows:

$$q_{{\mathrm{CO}}_{2}}=q_{{\mathrm{CO}}_{2}}^{max}\frac{C_{{\mathrm{CO}}_{2}}}{K_{{\mathrm{CO}}_{2}}+C_{{\mathrm{CO}}_{2}}}\frac{C_{NH_{4}^{+}}}{K_{NH_{4}^{+}}+C_{NH_{4}^{+}}}\frac{C_{\text{MH}}}{K_{\text{MH}}+C_{\text{MH}}}$$

(20) $$\left(1-\frac{C_{\text{Ac}}}{C_{\text{Ac}}^{*}}\right)\left(1-\frac{C_{\text{But}}}{C_{\text{But}}^{*}}\right)\left(1-\frac{C_{\text{Cap}}}{C_{\text{Cap}}^{*}}\right)$$

$$q_{\text{But}}^{elong}=q_{\text{But}}^{max}\frac{C_{\text{Ac}}-C_{\text{Ac}}^{t}w_{\text{Ac}}}{K_{\text{Ac}}+C_{\text{Ac}}-C_{\text{Ac}}^{t}w_{\text{Ac}}}Z_{\text{Ac}}\frac{C_{\text{MH}}}{K_{\text{MH}}+C_{\text{MH}}}$$

(21) $$\left(1-\frac{C_{\text{Ac}}}{C_{\text{Ac}}^{*}}\right)\left(1-\frac{C_{\text{But}}}{C_{\text{But}}^{*}}\right)\left(1-\frac{C_{\text{Cap}}}{C_{\text{Cap}}^{*}}\right)$$

$$q_{\text{Cap}}^{elong}=q_{\text{Cap}}^{max}\frac{C_{\text{Ac}}-C_{\text{Ac}}^{t}w_{\text{Ac}}}{K_{\text{Ac}}+C_{\text{Ac}}-C_{\text{Ac}}^{t}w_{\text{Ac}}}Z_{\text{Ac}}\frac{C_{\text{But}}-C_{\text{But}}^{t}w_{\text{But}}}{K_{\text{But}}+C_{\text{But}}-C_{\text{But}}^{t}w_{\text{But}}}$$

(22) $$Z_{\text{But}}\frac{C_{\text{MH}}}{K_{\text{MH}}+C_{\text{MH}}}\left(1-\frac{C_{\text{Ac}}}{C_{\text{Ac}}^{*}}\right)\left(1-\frac{C_{\text{But}}}{C_{\text{But}}^{*}}\right)\left(1-\frac{C_{\text{Cap}}}{C_{\text{Cap}}^{*}}\right)$$

### Simulation and Model Fitting Procedures

The kinetic maximum specific rates $q_{CO_{2}}^{max}$, $q_{\text{But}}^{max}$ and $q_{\text{Cap}}^{max}$ and the concentration thresholds $C_{Ac}^{t}$ and $C_{But}^{t}$ have not been experimentally determined in MES to date. Therefore, they were found by minimizing the residual sum of squares when fitting bulk concentrations of acetate, butyrate, and caproate over time. In this work, a residual is the difference between the experimental measurement from Jourdin et al. (2019) FTR2 reactor and the calculated value for that measurement obtained from the model. As the model is a non-linear system of equations, a non-linear least-squares regression was used. The minimization was performed using the Nelder-Mead method as implemented in MATLAB (Table 1).

## Results and Discussion

### Model Fitting

**FIGURE 2:**
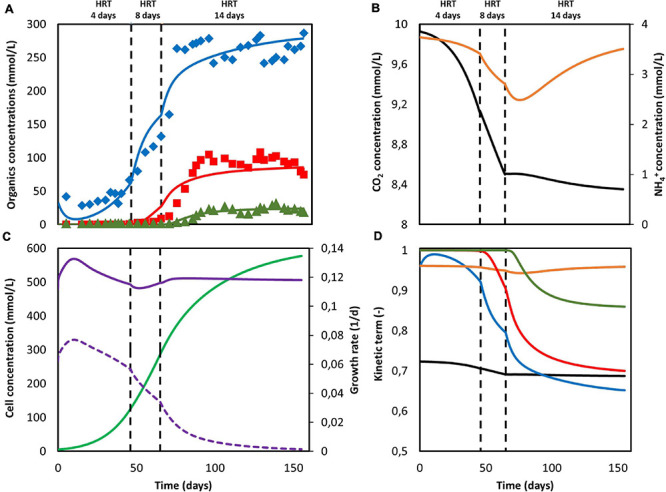
Model fitting results (lines) and experimental data Jourdin et al. (2019) (markers). **(A)** Concentration of acetate (blue), butyrate (red) and caproate (green); **(B)** Substrate concentration of CO_2_ (black) and NH_4_^+^ (orange); **(C)** Cell concentration (light green), maximum growth rate (purple), and growth rate (purple dashed line); **(D)** Kinetic uptake terms of CO_2_ (black) and NH_4_^+^ (orange), and kinetic product inhibition terms of acetate (blue), butyrate (red), and caproate (dark green). The vertical dashed lines represent the time when the hydraulic retention time was increased.

To obtain the necessary parameters for the kinetic equations, the model was fitted with the experimental results obtained by Jourdin et al. (2019). That system was operated in continuous mode with continuous CO_2_ sparging. Since the gas-liquid mass transfer coefficient *k_La_* for CO_2_ was not reported in the original work, its value was approximated from the reported inorganic carbon concentrations and found to be of the same order of magnitude as those reported on similar sparging mechanisms, i.e., 2.5 h^–1^ (Bajracharya et al., 2016). The hydraulic retention time (HRT), used to determine the dilution rate, was first increased from 4 to 8 days and then from 8 to 14 days. The best fitting results are shown in Figure 2A, together with the organics concentration measured experimentally by Jourdin et al. (2019). The model is able to follow the main trend of the experimental results. For the data points, the population standard deviation of the model was 31.7 mmol/L for acetate, 14.56 mmol/L for butyrate, and 3.65 mmol/L for caproate. The simulation shows deviations that can be attributed to the previously introduced simplifications on the model, such as exclusion of the dynamics occurring within a mixed culture. No special effect of increasing the HRT can be observed. The kinetic maximum specific rates $q_{CO_{2}}^{max}$, $q_{\text{But}}^{max}$, and $q_{\text{Cap}}^{max}$ and the concentration thresholds $C_{Ac}^{t}$ and $C_{But}^{t}$ were found to be −0.307 mol_CO2_ /(mol_*x*_ h), 2.12 × 10^–2^ mol_But_ /(mol_*x*_ h), 4.64 × 10^–3^ mol_Cap_ /(mol_*x*_ h), 123 mmol_Ac_/L and 43 mmol_But_/L, respectively. There is a lack of reported values in MES for these kinetic parameters, hence it is difficult to assess the values obtained here. Nagarajan et al. (2013) used a CO_2_ specific uptake rate on the same order of magnitude as the one obtained in this study, of −0.2 mol_CO2_ /(mol_*x*_ h), when characterizing acetogenic metabolism by using a genome-scale metabolic reconstruction approach; however, they failed to confirm the value experimentally (Figure 2).

The computed substrate concentrations over time (Figure 2B) show an initial decrease, with a later stabilization for CO_2_ and a slight increase for NH_4_^+^. This profile can be related to the developing cell population, with an initial exponential growth phase and a later plateauing when steady-state is reached (Figure 2C; Monod, 1949; Hwang et al., 2020). According to the model, cells use the nitrogen source for growth, whereas the carbon source is used for both growth, maintenance, and elongation reactions. The later ammonium concentration increase can then be attributed to biomass growth slowing down, and the stabilization of the carbon dioxide concentration to its continuous usage in maintenance and elongation reactions. The maximum growth rate (see Supplementary Information 2.1) was calculated to be on average 0.12 d^–1^ and within the range of typical reported growth rates for acetogens (0.1 to 0.4 d^–1^) (Ahring and Westermann, 1987; Klemps et al., 1987).

**FIGURE 3:**
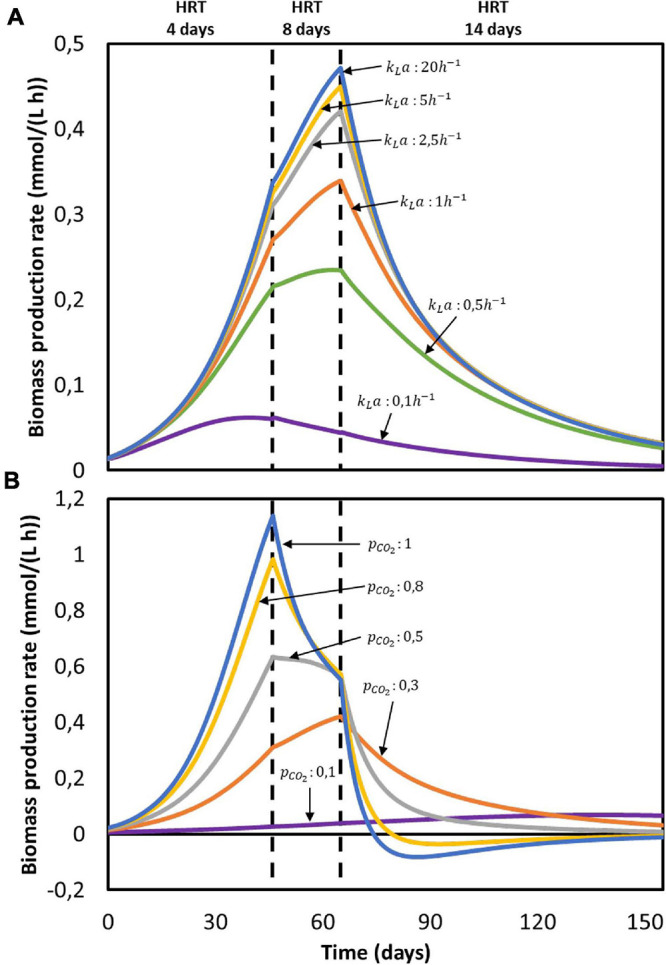
Computed biomass production rate in time for the reactor from Jourdin et al. (2019): **(A)** at different gas-liquid mass transfer coefficients (*k*_*L*_*a*) with *p*_*CO*_2__ = 0.3 and **(B)** at different gas CO_2_ partial pressures (*p*_*CO*_2__) with *k*_*L*_*a* = 2.5*h*^−1^. No experimental data available.

According to the model, none of the substrates was depleted. To study which factor is mainly controlling biological rates, the kinetic hyperbolic uptake and product inhibition terms from Eq. 20, 21, and 22 are graphically depicted over time in Figure 2D. These terms can be used as indicators for metabolic limitations, being responsible for the deviations between the theoretical maximum rates and the observed ones (Chen and Hashimoto, 1980; Pavlostathis and Giraldo-Gomez, 1991; Zeng and Deckwer, 1995). During the first 100 days of the experiment, carbon dioxide was the main factor limiting microbial kinetics, with a decrease of the maximum rate of about 30%. After day 100, product inhibition became the main limiting step, especially due to high acetate and butyrate concentrations. The initial carbon dioxide limitation can be attributed to its relatively high half-saturation constant of 3.8 mmol/L (Schuchmann and Müller, 2013), resulting on a fast drop of its uptake rate even when dissolved CO_2_ is still far from being depleted. A combination of poor gas-liquid mass transfer and a low gas inlet CO_2_ partial pressure were the limiting steps during this first period. The effect of changing these input parameters is depicted in Figure 3. Increasing the *k_La_* results on a higher biomass production rate, as CO_2_ is dissolved faster into the liquid (Figure 3A). This improvement decreases the higher the transfer coefficient. At a certain point, the rate of the gas-liquid mass transfer is sufficient to supply CO_2_ faster than what the microorganisms consume. Then, the uptake rate starts limiting the system. After an initial growth phase, all rates sharply decrease. This effect is induced by the increasing carboxylates concentrations and the subsequent product inhibition on cell metabolism (Supplementary Figures 2A–C in the Supplementary Information 2.2). The CO_2_ partial pressure (p_CO__2_) of the feed gas determines the saturation concentration at which carbon dioxide can be dissolved into the liquid (Weiss, 1974). Increasing this partial pressure substantially improves biomass production rate, as shown in Figure 3B. In this case, the positive effect is because of a higher driving force for gas-liquid mass transfer, i.e., the equilibrium concentration of CO_2_ with a p_CO__2_ of 1 is of 34 mmol/L, three times higher than with a partial pressure of 0.3. The higher the partial pressure of CO_2_ used the more pronounced the effect of product inhibition is. This can be attributed to the microbial dynamics during the initial part of the run, reaching the carboxylates’ inhibiting concentrations at a faster rate with higher CO_2_ fraction in the inlet gas (Supplementary Figures 2D–E in the Supplementary Information 2.2) (Figure 3).

### Model Validation: Prediction vs. Experimentation

A wide range of biological systems and operational conditions applied to MES are described in literature. In this paper, and in order to study the prediction capabilities of the model, the work of Jourdin et al. (2018) and Marshall et al. (2013) were chosen because of their detailed experimental descriptions and model input parameters availability. The model structure is largely the same as in the fitting case previously discussed. Any model parameters modification done to reflect differences between the studied systems can be found in the Supplementary Informations 2.3, 2.4.

#### CO_2_ Supply Strategy Greatly Impacts Reactor Performance

**FIGURE 4:**
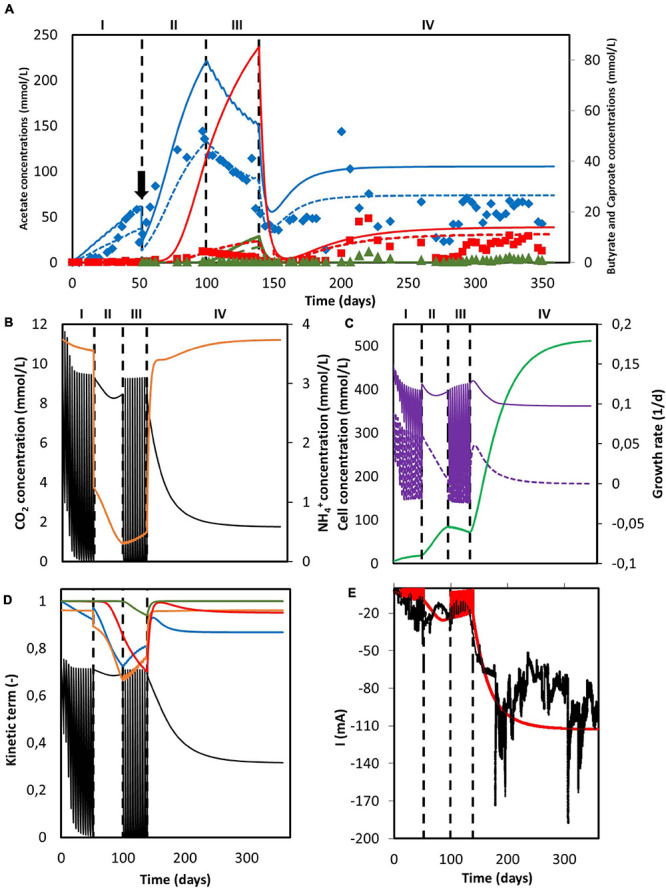
Simulation results of a MES system with changing feed strategies and operational modes. **(A)** Predicted concentration of acetate (solid blue line), n-butyrate (solid red line) and n-caproate (solid dark green line) and experimental data from Jourdin et al. (2018) of acetate (blue diamonds), n-butyrate (red squares), and n-caproate (dark green triangles). Adjusted concentrations with reported electron recoveries are shown with dashed lines for acetate (blue), n-butyrate (red), and n-caproate (dark green); **(B)** Concentration of CO_2_ (black) and NH_4_^+^ (orange); **(C)** Microbial cell concentration (light green), calculated maximum growth rate (purple), and growth rate (purple dashed line); **(D)** Kinetic hyperbolic uptake terms of CO_2_ (black) and NH_4_^+^ (orange), and kinetic product inhibition terms of acetate (blue), n-butyrate (red), and n-caproate (dark green); **(E)** Predicted (red) and experimental (black) current. The vertical dashed lines represent the time when reactor operation was switched from fed-batch to batch and from batch to continuous mode. The black arrow indicates when substantial leakage of the medium occurred.

Simulation results for the system utilized by Jourdin et al. (2018) can be found in Figure 4. The reactor was operated in fed-batch mode under a discontinuous CO_2_ sparging regime during periods I and III, in batch mode with continuous sparging of CO_2_ during period II and in continuous mode with continuous sparging of CO_2_ during period IV. When comparing the organics concentration obtained from the simulation with the experimental data, although showing a similar trend, the model predictions deviate from the experimental results (Figure 4A). Calculated acetate and n-butyrate concentrations are substantially higher, especially during periods II and III. For the data points, the population standard deviation of the model was 50.11 mmol/L for acetate, 10.79 mmol/L for butyrate, and 1.13 mmol/L for caproate.

This overshooting contrasts with the good description by the model of the current consumption, as can be seen in Figure 4E. This good match between simulation and experimental data on the electron consumption, together with the mismatch on organics prediction indicates an overestimation of the coulombic efficiency. These differences could be attributed to hydrogen production, as Jourdin et al. (2018) reported electron recoveries that ranged from 20% to 70% during the first three periods of the run and from 60% to 100% during the last period. This dynamic behavior between carboxylates production and hydrogen evolution is not included in the present model. During the fitting in section “Model Fitting”, the biological rates from the model were calculated to match the organics concentration evolution over time from the experimental results. As a consequence, the coulombic efficiency of the system leading to electron losses was not taken into account. When the experimentally reported recoveries are applied to the organics concentration predicted by the model, a better representation of the experimental data is obtained (dashed lines in Figure 4A). The population standard deviation of the adjusted model was 32.09 mmol/L for acetate, 3.62 mmol/L for butyrate, and 0.68 mmol/L for caproate. This highlights that the model presented in this paper is able to predict the performance of a MES system with a 100% coulombic efficiency, giving for a certain set of conditions an approximation of what the best possible outcome can be (Figure 4).

As can be seen in Figure 4B, carbon dioxide was periodically depleted in periods I and III as the multiple sparging periods were not able to keep up with its consumption rate. When the feeding strategy was changed to continuous addition in period II, an initial decrease with a later slight increase of CO_2_ concentration is observed, but no depletion occurred. The later increase of the CO_2_ concentration can be attributed to the plateauing of the biomass concentration (see Figure 4C; Hwang et al., 2020), as the model shows that the ammonium concentration was continuously decreasing and close to being depleted. No nitrogen limitation was observed. Ammonium concentration slightly decreased but stayed high during period I, severely decreased in period II and increased during period III. This later increase can be attributed to the carbon dioxide depletion and the subsequent halt of cell growth, inducing cell death and a decrease of the biomass concentration (Figure 4C; Váchová and Palková, 2005). During period IV, when the operational mode was switched from batch to continuous, i.e., nutrients and CO_2_ were continuously added, CO_2_ concentration shows an initial decrease with a later stabilization when the steady-state is reached. On the other hand, NH_4_^+^ concentration peaks at the beginning and then slowly stabilizes. This sharp increase is attributed to the accumulation produced by the constantly added fresh medium.

The depletion of carbon dioxide during fed-batch periods completely stopped the growth metabolism of the cells (Figure 4D). Even though the nitrogen source could be expected to become the bottleneck of the system during period II, the uptake of CO_2_ was still the rate limiting microbial kinetics. This can be attributed to the low ammonium half-saturation constant, buffering the effect of a low concentration on the overall kinetics. Again, when the operational mode was switched to continuous in period IV and the steady-state was reached, the CO_2_ uptake term was the limiting factor, decreasing the maximum rate by about 65%. In summary, it is clear that the limiting step during the entire duration of Jourdin’s experiment was the supply of carbon dioxide, pointing out to the importance of not only the amount of CO_2_ added but also how this addition is carried out.

#### Continuous Operation Benefits Biofilm Growth

**FIGURE 5:**
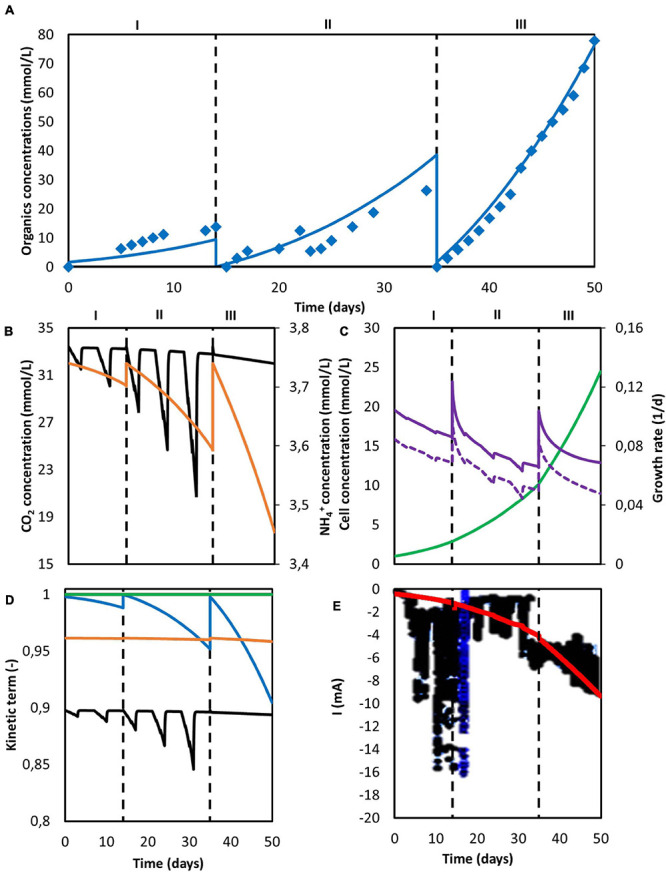
Computed simulation results of a MES system with three consecutive batches. **(A)** Predicted concentration of acetate (blue line) and experimentally determined acetate concentration from Marshall et al. (2013) (blue diamonds); **(B)** Concentration of CO_2_ (black) and NH_4_^+^ (orange); **(C)** Microbial cell concentration (light green), calculated maximum growth rate (purple), and growth rate (purple dashed line); **(D)** Kinetic hyperbolic uptake terms of CO_2_ (black) and NH_4_^+^ (orange), and kinetic product inhibition term of acetate (blue); **(E)** Predicted (red) and experimental (black) current. The vertical dashed lines represent the three different batch operations.

A second simulation to reproduce the set of data obtained by Marshall et al. (2013) was performed, and the results obtained from the model are showed in Figure 5. A series of consecutive batches was simulated. After every batch, the catholyte was replaced by fresh medium while the biomass remained attached to the electrode material. The first two batches (periods I and II) operated under a discontinuous CO_2_ sparging regime, whereas the third batch (period III) was continuously sparged with pure CO_2_. The model properly predicts the acetate concentration profile obtained experimentally (Figure 5A). For the data points, the population standard deviation of the model was 1.94 mmol/L for acetate. The elongation thresholds from Eq. 15 and Eq. 16 allowed to properly reproduce a system where only acetate was produced (Figure 5).

Carbon dioxide concentration oscillated during the intermittent sparging phases in periods I and II and showed a subtle decrease during period III, but was far from being depleted (Figure 5B). However, it should be stressed that the actual *k_La_* of their sparging method was not reported and thus assumed to be the same as in Jourdin et al. (2019). Therefore it is difficult to conclude the system was not CO_2_ limited. NH_4_^+^ concentration showed a batch-like behavior, decreasing faster in every consecutive batch. This is attributed to the increasing biomass concentration and its exponential behavior, as according to the model nitrogen consumption is strictly bounded to microbial growth (Figure 5C). Although nitrogen uptake rate was exponentially increasing, NH_4_^+^ was far from depletion.

**FIGURE 6:**
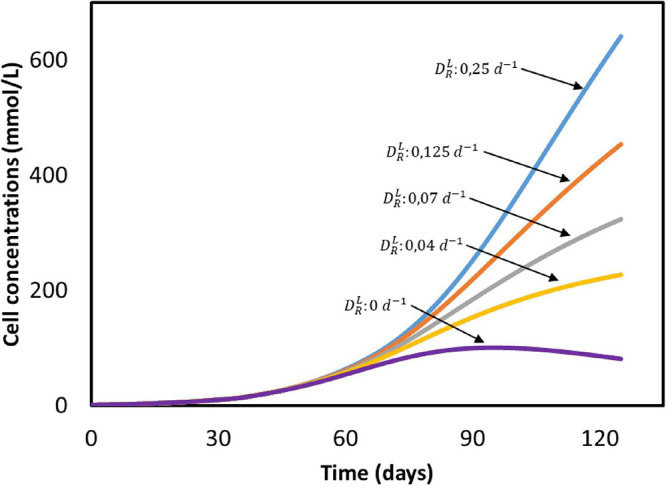
Cell concentrations in time at different dilution rates ($D_{R}^{L}$) based on the system from Marshall et al. (2013). All input parameters are the same between simulations, with the exception of the dilution rate. No experimental data available.

The biomass concentration obtained after 50 days (25 mmol/L) is five times lower than the amount produced in a continuous reactor with constant CO_2_ sparging (125 mmol/L), as can be seen when comparing Figures 2C, 5C. The effect of the dilution rate on the biomass growth is shown in Figure 6. The higher biomass concentration achieved with biofilm-driven systems operating in continuous mode can be attributed to the exchange flow. Since microorganisms grow attached to the electrode, and are therefore not affected by this dilution rate, the difference in growth rate is caused by the other dilute species concentrations in the system. In continuous operation, nutrients are constantly replenished while products are removed from the reactor, diminishing the effects of low substrate concentrations and product inhibition (Michel-Savin et al., 1990; Andrić et al., 2010). However, in a batch system like the one used by Marshall et al. (2013), nutrients deplete and products accumulate faster over time.

The kinetic parameters are presented in Figure 5D. Carbon dioxide uptake was the limiting kinetic rate during all three batches. During the last intermittent sparging phase in period II a total decrease of the maximum uptake rate up to 15% was reached, but stabilized at 10% in period III when continuous gas sparging was applied. Although a 30% decrease on the carbon concentration during period II is observed in Figure 5B, the use of pure CO_2_ by Marshall helped to mitigate the effect on the microbial kinetics. The amount of carbon dioxide that can be dissolved in the system increases linearly with the CO_2_ partial pressure used in the gas (Weiss, 1974). A 3.3 times higher CO_2_ liquid concentration was obtained by the use of 100% CO_2_ as feeding gas when compared with the 30% used by Jourdin et al. (2018). This higher concentration mitigated the decrease of the hyperbolic term for CO_2_ uptake and avoided a more severe rate inhibition by the carbon source. Since the limiting effect of CO_2_ and other chemical species in microbial kinetics is low, it is likely that the reactor was limited by the absolute amount of biomass in the system.

The current in periods II and III is correctly described by the model, as can be seen in Figure 5E. However, during the first period, predictions substantially deviate from the experimental values, with the calculated ones being lower than the ones observed by Marshall. Again, this deviation can be attributed to hydrogen and other by-products formation, as side-reactions are not accounted for in the current model. Microbial attachment and biofilm formation can be a slow process in MES systems, as bacteria do not obtain much energy from CO_2_ reduction (Schuchmann and Müller, 2014). Hence, it is likely the case that during the first batch, as the biomass was starting to colonize the cathode, electrons were redirected toward hydrogen evolution. When cell concentration further increased, these electrons started being used in microbial reactions instead, giving the initial increase in the cathodic current observed at the beginning of period II (Figure 6).

### Model Assumptions Evaluation

#### Concentration Gradients Over the Reactor

The model assumes no concentration gradients in the individual domains. Hence, concentration steps occur only between the domains or at the inflow. To support the assumption that gradients are negligible, the magnitude of the concentration steps will be discussed. Experimental measurements were performed at the sampling port (*S* in Figure 1B), thus computed concentrations refer to those leaving the second bulk liquid compartment ($C_{i}^{B2}$). Individual mass balances over each domain, together with an explanation on how the concentration gradients were calculated can be found in the Supplementary Information 3.1. Taking into consideration all compounds present in the system, carbon dioxide and protons are the ones expected to have the highest concentration steps along the reactor.

**FIGURE 7:**
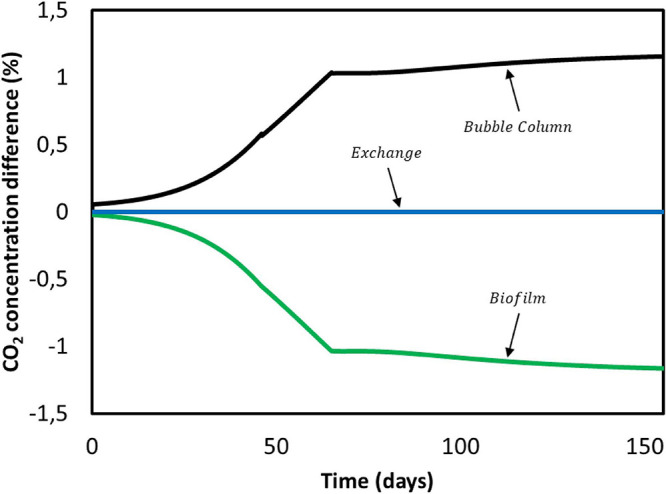
CO_2_ concentration difference between domains during Jourdin et al. (2019) experiment.

First, we focus on carbon dioxide. It is consumed by the microorganisms in the biofilm domain and transfers from the gas phase to the bulk liquid in the bubble column. Results obtained for CO_2_ concentration gradients over the entire duration of Jourdin et al. (2019) experiment are shown in Figure 7. Positive values indicate a concentration increase between the previous and the current compartment, whereas a negative value refers to consumption. In no case the concentration difference exceeds ± 1.5%. This is attributed to the small ratio between the dilution flow rate of 0.018 mL/min and the recirculation rate of 200 mL/min. The characteristic CO_2_ reaction time is estimated to be 4 min, while the residence time of the convective flow is 0.13 min (Supplementary Information 3.2). The ratio between these times causes overall concentration changes in the system to become significant after multiple recirculations rather than after a single pass through the biofilm domain. Therefore, for the purpose of this model CO_2_ concentration gradients in the reactor domains can be neglected (Figure 7).

#### pH at the Biofilm

The pH of a biologically active cathodic chamber is highly dependent on the acid-base reaction equilibria. CO_2_ and all products accounted for in the current model behave as acid. In addition, the presence of a buffer must also be taken into consideration. It is therefore the balance between these production and consumption processes, that determines local pH gradients. In the present study, pH was assumed to be constant but since it has a great influence on both electrochemical and biological reactions, this assumption needs to be further investigated.

Bulk pH is strictly monitored and controlled by acid and base addition, therefore gradients due to protons diffusing from anode to biofilm can be neglected. However, reactions are happening within the biofilm and not in the liquid bulk, therefore a gradient might still be present at the vicinity of the electrode. An estimation of both characteristic reaction and diffusion times for CO_2_, H^+^, and the buffer compound was used to determine if large pH gradients would be present in the biofilm. These calculations and respective explanations can be found in the Supplementary Information 3.2. Results indicate that although H^+^ ions are not able to diffuse into the biofilm quick enough, the presence of a buffer allows to compensate for the consumed H^+^ in the biofilm by a buffering reaction with a reaction time in the same order of magnitude as for CO_2_. According to the calculated diffusion and reaction times, CO_2_ diffusion slowed down the biological reaction, which at the same time limited the proton consumption rate by the microorganism. The protonated buffer compound diffusion through the biofilm and buffering reaction would then have to keep up to effectively control pH. By analogy to the case treated by Vander Wielen et al. (1997), the mentioned decrease in the general metabolic rate may have been sufficient to allow the buffer to prevent large pH gradients. A real biofilm is not homogeneous, and large gradients might occur at conditions different from those simulated here. Experimental data on pH values throughout the biofilm are required to validate the calculations.

#### Potential Model Improvements

Only one bacterial population has been included in this model, even though the simulated reactors were systems working with mixed cultures. Therefore, the model could be expanded to include multiple microorganisms with differentiated metabolisms (Rauch et al., 1999; Xavier et al., 2005). As an example, solventogenesis (i.e., ethanol production from acetate) and chain elongation could be described independently from acetogenesis, allowing a deeper investigation of possible interactions between intermediate compounds, substrates, and microorganisms. It could also lead to a better understanding of which metabolic routes are being used by microorganisms to reduce CO_2_ into longer chain products such as caproate.

As previously described, pH gradients and other chemicals’ gradients should be further investigated for biofilm-driven systems. The addition of acid-base equilibria, hydrogen evolution, and electromigration would allow to better understand gradients at the biofilm level, potentially giving additional insights on rate limiting processes. These gradients could also help to understand biofilm development and biofilm/planktonic cells dynamics. Moreover, mass and ion transport can be expected to become of paramount importance as MES current density and microbial productivity continue to increase (Jourdin and Burdyny, 2020). In this sense, extending the model to a multi-dimensional model such as the ones developed in Picioreanu et al. (2007), Picioreanu et al. (2010), Bottero et al. (2013) for microbial fuel cells could be interesting for this purpose.

The use of a product inhibition model is necessary to account for the inherent toxicity of the produced carboxylates. Even though the model used in this paper gave good results, it should be expanded and validated with experiments in which products have been added to the inflow. This would give a better and more tailored description of product toxicity within these systems, and subsequently a better understanding of chain elongation metabolism and kinetics.

### Model Implications

Simulations done in this work suggest that CO_2_ can limit the rate of microbial electrosynthesis. Carbon dioxide is the main substrate and the only carbon source in most MES reactors, hence its concentration has a great impact on cell kinetics. Its relatively high half-saturation constant and low solubility make microorganisms very susceptible to small changes in its dissolved concentration. The model indicates that the use of pure CO_2_ as feeding gas can mitigate this effect, as shown experimentally in Rojas et al. (2021). However, it is important to note here that avoiding a kinetic limitation may not be enough to substantially increase productivity, since CO_2_ diffusion might become the limiting step at some point. Results also indicate not to underestimate the critical effect of the CO_2_ delivery strategy on reactor performance (Izadi et al., 2020). In MES studies, mass transfer coefficients are hardly ever reported, therefore it is difficult to conclude that poor CO_2_ delivery systems are one of the reasons why obtained production rates are still low across the field (Prévoteau et al., 2020). Regardless of the supply method used, its mass transfer capability should always be assessed. The model developed in this work can be used to determine the minimum mass transfer capability required to avoid kinetic limitations by the supply method. By ensuring that the used system is able to deliver enough CO_2_ to sustain a highly active microbial population, a better understanding can be achieved of which steps are intrinsically limiting steps in said MES processes.

To date, most MES studies have been performed under batch conditions and not many researchers used a continuous reactor for reducing CO_2_ (Batlle-Vilanova et al., 2016; Arends et al., 2017; Bajracharya et al., 2017; LaBelle and May, 2017; Molenaar et al., 2017; Jourdin and Burdyny, 2020). The model indicates that the continuous mode enhances cell growth, hence it might be one of the reasons why dense biofilms have been mainly obtained with this type of reactors. This can be attributed to a selective pressure that benefits attached cells since under a continuous operation, planktonic populations are easily washed out the reactor. However, biofilm development is subject to multiple parameters, and the operational mode is just one of them. It has to be noted that increasing the capacity for growth of a bacterial population does not necessarily mean that the culture will be able to grow that much. As an example, in biofilm-driven systems the electrode surface area available for attachment and its roughness are also key parameters that limit the development of a thick biofilm (Myint et al., 2010; Ammar et al., 2015). In that sense, the model can be used to calculate the maximum cell growth that can be obtained with a certain system under a specific set of operational conditions.

## Conclusion

The mathematical model presented in this work is able to accurately describe the behavior of different biofilm-driven MES reactors operating in batch, fed-batch, and continuous mode. It was found that under previously reported operational conditions biomass growth was partially limited by the CO_2_ dissolved concentration. This implies that a more careful assessment of the inorganic carbon supply method is needed to increase production rates. Furthermore, simulations show that operating in continuous mode leads to higher cell densities. Since most current studies are done in batch mode, this might be one of the reasons why cell titers are far below their theoretical maximum (Jourdin and Burdyny, 2020; Prévoteau et al., 2020). These results demonstrate the value of such models in understanding MES systems, and highlight their usefulness when analyzing current process limitations.

## Data Availability Statement

The original contributions presented in the study are included in the article/Supplementary Material, further inquiries can be directed to the corresponding author/s.

## Author Contributions

OC-P developed the model and drafted the manuscript. AS and LJ contributed to the modeling effort and data interpretation. All authors contributed to manuscript revision, conception, design of the study, read and approved the submitted version.

## Conflict of Interest

The authors declare that the research was conducted in the absence of any commercial or financial relationships that could be construed as a potential conflict of interest.
