# Supplementary material for: A General Model for Biofilm-Driven Microbial Electrosynthesis of Carboxylates From CO2
Source: Front Microbiol. 2021 Jun 4;12:669218. doi: 10.3389/fmicb.2021.669218 (PMC8211901; doi:10.3389/fmicb.2021.669218)
Supplement: Supplementary file 1 [file Data_Sheet_1.docx]

**A general model for biofilm-driven microbial electrosynthesis of carboxylates from CO_2_**

**Oriol Cabau-Peinado^1^, Adrie J.J. Straathof ^1^, Ludovic Jourdin^1*^**

^1^Department of Biotechnology, Faculty of Applied Sciences, Delft University of Technology, Delft, The Netherlands

***Supplementary Information***

1. **Model description**
   1. **Batch H-cell reactor from Marshall et al. (2013)**


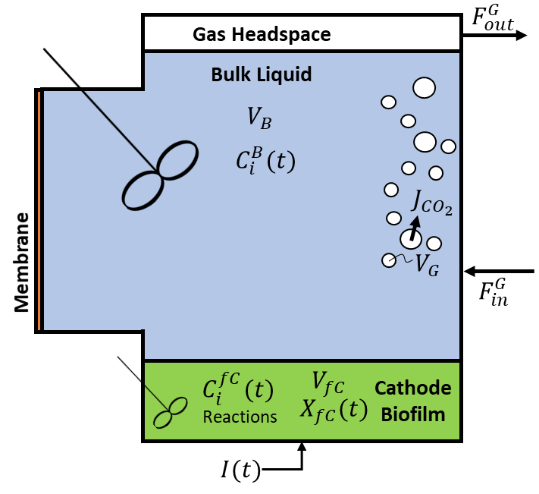


**Figure S1.** Model domains of the batch reactor based on Marshall et al. (2013).

- 1. **Mass balances of the dissolved chemical species**

***Continuous reactor from Jourdin et al. (2019) and Jourdin et al. (2018)***

Overall concentration changes in time of all dissolved species (*i*) are caused by biological reactions, electrochemical reactions, gas/liquid CO_2_ transfer and the exchange of medium (e.g. continuous operation). The total volume of the reactor ($V_{T}^{C}$), including the bioelectrochemical cells and the bubble column, is described in Eq. S1. In a continuous operating system, a dilution rate term ($D_{T}^{L}$) can be defined as shown in Eq. S2. This rate describes the relation between the exchange flow of fresh medium into the system ($F_{in}^{L}$) and the volume of said system. It can also be expressed as the inverse of the retention time ($HRT$).

$$\begin{aligned} V_{T}^{C}=V_{B1}+V_{B2}+V_{fc}+V_{BC} \#\left( S1 \right) \end{aligned}$$

$$\begin{aligned} D_{T}^{L}=\frac{F_{in}^{L}}{V_{T}}=\frac{1}{HRT}\#\left( S2 \right) \end{aligned}$$

The mass balance equations over the entirety of the reactor volume can be described as:

$$\begin{aligned} \frac{{dC}_{i}}{dt}=D_{T}^{L}\left( C_{i}^{in}-C_{i} \right)+\frac{V_{fc}}{V_{T}^{C}}r_{i}^{fc}\pm\frac{V_{fc}}{V_{T}^{C}}r_{M}^{elec}+\frac{V_{BC}}{V_{T}^{C}}k_{L}a\left( C_{{CO}_{2}}^{*}-C_{{CO}_{2}}^{BC} \right)\#\left( S3 \right) \end{aligned}$$

The set of equations obtained from Eq. S3 includes changes produced by the continuous operation mode ($D_{T}^{L}$), reactions within the biofilm ($r_{i}^{fc}$), the electrochemical reaction of the mediator pair ($r_{M}^{elec}$) and the gas/liquid CO_2_ mass transfer ($k_{L}a$). Volume corrections to account for the difference in volume between compartments are also included. The initial conditions are $C_{i}\left( 0 \right)=C_{i}^{0}$.

***Batch reactor from Marshall et al. (2013)***

The main difference between the H-cell reactor and the reactor from Jourdin et al. consists on the operating mode and the different liquid compartments. The total volume of the reactor ($V_{T}^{H}$) is now the sum of the bulk and the cathode/biofilm volumes. Marshall et al. (2013) operated their reactor in batch mode, meaning no exchange flow was applied, hence $D_{T}^{L}=0$.

$$\begin{aligned} V_{T}^{H}=V_{B}+V_{fc} \#\left( S4 \right) \end{aligned}$$

$$\begin{aligned} \frac{{dC}_{i}}{dt}=\frac{V_{fc}}{V_{T}^{H}}r_{i}^{fc}\pm\frac{V_{fc}}{V_{T}^{H}}r_{M}^{elec}+\frac{V_{B}}{V_{T}^{H}}k_{L}a\left( C_{{CO}_{2}}^{*}-C_{{CO}_{2}}^{B} \right) \#\left( S5 \right) \end{aligned}$$

- 1. **Gibbs energies correction**

The Gibbs energies of reaction ${\Delta G}_{Cat}^{0}$, ${\Delta G}_{An}^{0}$, ${\Delta G}_{But}^{0}$ and ${\Delta G}_{Cap}^{0}$ were first corrected for temperature using the Gibbs-Helmholtz equation:

$$\begin{aligned} {\Delta G}_{i}^{0T}={\Delta G}_{i}^{0}\frac{T}{T_{0}}+{\Delta H}_{i}^{0}\frac{T_{0}-T}{T_{0}} \#\left( S6 \right) \end{aligned}$$

Then, the concentration-dependent free energy change was calculated for every reaction. The effect of non-ideality was neglected, hence the activity coefficients of all compounds were assumed to be equal to 1. Proton concentrations ($C_{H^{+}}={10}^{-5.8} mol L^{-1}$) were assumed constant during all simulations.

$$\begin{aligned} {\Delta G}_{i}^{01}={\Delta G}_{i}^{0T}+RT\sum_{j=1}^{n} \left( ln\left( C_{j} \right)\cdot Y_{j}^{i} \right) \#\left( S7 \right) \end{aligned}$$

- 1. **Redox mediator pair**

Microbial metabolism is a complex network of interlinked biochemical reactions. Different redox mediator couples are used for multiple reactions, e.g., NADH/NAD^+^, H_2_/H^+^, or Fd_(red)_/Fd_(ox)_. It is known that some intermediate reactions within the anaerobic reduction of CO_2_ to acetate require electrons at low potential, likely at that of ferredoxin (Furdui and Ragsdale, 2000; Kracke et al., 2016). However, NADH is also known to be involved in the energy conservation mechanisms of acetogenic bacteria (Bertsch et al., 2015). Moreover, the anaerobic chain elongation of acetate to carboxylates has been seen to be highly dependent on NADH (Spirito et al., 2014). Therefore, it is not straightforward which mediator pair limits microbial metabolism in MES.

In this work, a black box organism is assumed and therefore, intermediate reactions are not accounted for during the thermodynamic state analysis calculations (Kleerebezem and Van Loosdrecht, 2010). For modelling purposes, the energy gain by the microorganism was simplified into one reaction, i.e. oxidation of the electrochemically reduced mediator pair (Eq. 11 in the main text). Hence, from a pure energetic point of view, any of the previously introduced mediators could be used. The proposed model only allows the use of one mediator pair, and since a mixed microbial biofilm is simulated it is not possible to know what redox couple would be limiting biological rates. In this study, as the NADH/NAD^+^ pair is one of the most common redox couples in microbial metabolisms (Madigan et al., 1997), all simulations are performed with the standard redox potential of NADH/NAD^+^ of -320 mV vs SHE (Schuchmann and Müller, 2014).

1. **Results**
   1. **Calculation of the maximum growth rate**

In this paper, the maximum growth rate is approximated by neglecting the effect of substrate concentrations and products inhibition. It is therefore calculated as follows:

$$\begin{aligned} \mu^{max}=\frac{q_{{CO}_{2}}^{max}+m_{{CO}_{2}}}{Y_{{CO}_{2}}^{Met}} \#\left( S8 \right) \end{aligned}$$

- 1. **Simulated product inhibition at different** $\boldsymbol{k}_{\boldsymbol{L}}\boldsymbol{a}$ **and** $\boldsymbol{p}_{\boldsymbol{CO}_{\boldsymbol{2}}}$ **in the system from Jourdin et al. (2019)**


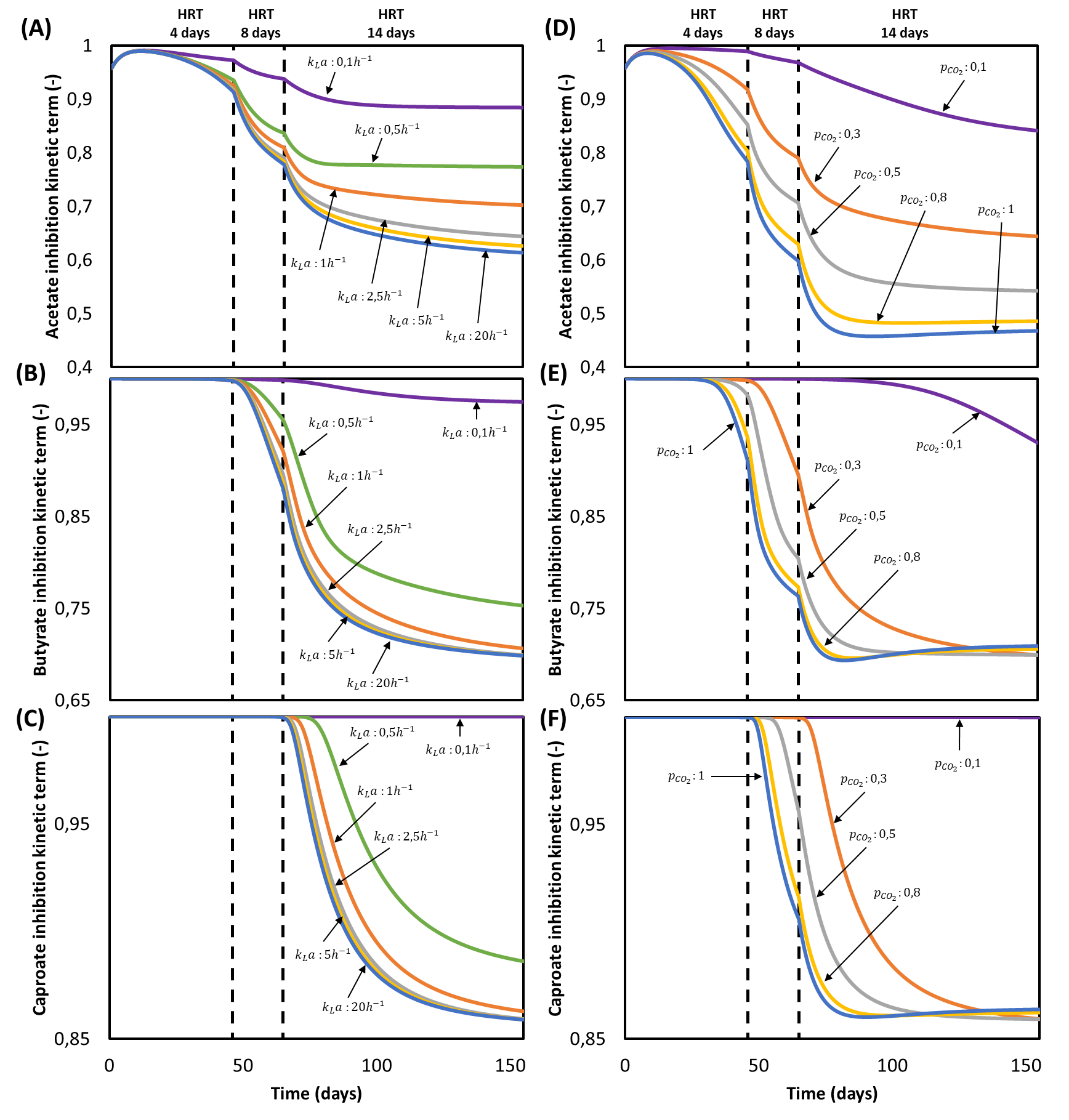


**Figure S2.** Simulated kinetic product inhibition terms of acetate, butyrate, and caproate in time for the reactor from Jourdin et al. (2019): **(A-C)** at different gas-liquid mass transfer coefficients ($k_{L}a$) with $p_{{CO}_{2}}=0.3$ and **(D-E)** at different gas CO_2_ partial pressures ($p_{{CO}_{2}}$) with $k_{L}a=2.5h^{-1}$.

- 1. **Model validation Jourdin et al. (2018)**

All parameters used in simulations were taken from **Table 1** found in the main text, with the exception of the values presented in **Table S1**.

**Table S1.** Parameters used to represent the reactor from Jourdin et al. (2018).

| Parameter | Symbol | Value | Units | Source |
| --- | --- | --- | --- | --- |
| *Bulk liquid* |  |  |  |  |
| Dilution rate | $D_{R}^{L}$ | Variable | 1/d | (Jourdin et al., 2018) |
| Gas-liquid mass transfer coefficient | $k_{L}a$ | 0.45 | 1/h | Calculated from (Jourdin et al., 2018) |
| Acetate initial concentration | $C_{Ac}^{0}$ | 0 | mol/m^3^ | (Jourdin et al., 2018) |

- 1. **Model validation Marshall et al. (2013)**

All parameters used in simulations were taken from **Table 1** found in the main text, with the exception of the values presented in **Table S2**.

**Table S2.** Parameters used to represent the rector from Marshall et al. (2013).

| Parameter | Symbol | Value | Units | Source |
| --- | --- | --- | --- | --- |
| *Bulk liquid* |  |  |  |  |
| Bulk liquid volume | $V_{B}$ | 135 | mL | (Marshall et al., 2013) |
| Dilution rate | $D_{R}^{L}$ | 0 | 1/d | (Marshall et al., 2013) |
| H^+^ concentration | $C_{H^{+}}^{B}$ | Variable per batch | mol/L | (Marshall et al., 2013) |
| Gas-liquid mass transfer coefficient | $k_{L}a$ | 0.45 | 1/h | Chosen |
| Acetate initial concentration | $C_{Ac}^{0}$ | 0 | mol/m^3^ | (Marshall et al., 2013) |
|  |  |  |  |  |
| *Biofilm* |  |  |  |  |
| Biocathode volume | $V_{fc}$ | 15 | mL | (Marshall et al., 2013) |
| Initial biomass concentration | $C_{X}^{0}$ | 1 | mol/m^3^ | Chosen |

1. **Critical assumptions evaluation**
   1. **Gradients over the reactor**

Concentration changes in time of all dissolved species (*i*) for all four compartments are:

| $\begin{aligned} \frac{dC_{i}^{B1}}{dt}=\frac{F_{R}^{L}}{V_{B1}}\left( C_{i}^{B1,in}-C_{i}^{B1} \right) \left( S9 \right) \end{aligned}$ | $\begin{aligned} \frac{dC_{i}^{B2}}{dt}=\frac{F_{R}^{L}}{V_{B2}}\left( C_{i}^{fc}-C_{i}^{B2} \right) \left( S11 \right) \end{aligned}$ |
| --- | --- |
| $\begin{aligned} \frac{dC_{i}^{fc}}{dt}=\frac{F_{R}^{L}}{V_{fc}}\left( C_{i}^{B1}-C_{i}^{fc} \right)+r_{i}^{fc} \left( S10 \right) \end{aligned}$ | $\begin{aligned} \frac{dC_{i}^{BC}}{dt}=\frac{F_{R}^{L}}{V_{BC}}\left( C_{i}^{B2}-C_{i}^{BC} \right)+k_{L}a\left( C_{{CO}_{2}}^{*}-C_{{CO}_{2}}^{BC} \right) \left( S12 \right) \end{aligned}$ |

Reactions in the bulk liquid are negligible, hence any change occurring in the bulk compartment 2 has to come from the biological reactions occurring in the cathode/biofilm compartment. Combining Eq. S10 and S11, an expression can be derived for the concentration in the first bulk domain.

$$\begin{aligned} \frac{dC_{i}^{B2}}{dt}=\frac{dC_{i}^{fc}}{dt} \#\left( S13 \right) \end{aligned}$$

Since no reaction occurs in the bulk liquid, $C_{i}^{B2}=C_{i}^{fc}$ hence Eq. S13 becomes zero and the following equation is obtained:

$$\begin{aligned} C_{i}^{B1}=C_{i}^{B2}-\frac{V_{fc}}{F_{R}^{L}}r_{i}^{fc} \#\left( S14 \right) \end{aligned}$$

A similar balancing can be done to obtain the concentration after the bubble column. By combining Eq. S11 and S12, and taking into consideration that $C_{i}^{B2}=C_{i}^{fc}$, the expression for $C_{i}^{BC}$ can be obtained. As expected, this expression is only valid for CO_2_ since the other dissolved species concentrations remain constant.

$$\begin{aligned} C_{i}^{BC}=C_{i}^{B2}+\frac{V_{BC}}{F_{R}^{L}}k_{L}a\left( C_{{CO}_{2}}^{*}-C_{{CO}_{2}}^{BC} \right) \#\left( S15 \right) \end{aligned}$$

The concentration change due to the exchange flow is:

$$\begin{aligned} \frac{{dM}_{i}^{B1,in}}{dt}=F_{R}^{L}C_{i}^{BC}+F_{in}^{L}C_{i}^{L,in}-F_{out}^{L}C_{i}^{BC}-F_{R}^{L}C_{i}^{B1,in}\#\left( S16 \right) \end{aligned}$$

Following previous reasoning, the change in the bulk liquid 1 compartment is equal to the change due to the addition of fresh medium meaning $C_{i}^{B1,in}=C_{i}^{B1}$. Thus, combining Eq. S9 and S16:

$$\begin{aligned} C_{i}^{B1,in}=C_{i}^{BC}\left( 1-\frac{F_{out}^{L}}{F_{R}^{L}} \right)+C_{i}^{L,in}\frac{F_{in}^{L}}{F_{R}^{L}} \#\left( S17 \right) \end{aligned}$$

The concentration difference between two consecutive compartments can then be calculated as follows:

$$\begin{aligned} {\%}_{i}^{Difference}=\frac{C_{i}^{Compartment 2}-C_{i}^{Compartment 1}}{C_{i}^{Compartment 2}}\cdot100 \#\left( S18 \right) \end{aligned}$$

- 1. **pH at the biofilm**

The characteristic reaction time and diffusion time can be calculated with Eq. S19 and Eq. S20, respectively (Vander Wielen et al., 1997). Concentrations and volumetric rates were taken from the steady state of Jourdin et al. (2019) reactor. Results can be found in **Table S3** and the parameters used in **Table S4**.

$$\begin{aligned} Reaction time=\frac{C_{i}^{liquid}}{r_{i}^{b}} \#\left( S19 \right) \end{aligned}$$

$$\begin{aligned} Diffusion time=\frac{{L_{b}}^{2}}{D_{i}} \#\left( S20 \right) \end{aligned}$$

**Table S3.** Characteristic times calculated for the steady state in Jourdin et al. (2019).

| Chemical species | Reaction time | Diffusion time | Units |
| --- | --- | --- | --- |
| *CO_2_* | 4.2 | 2.2 | min |
| *H^+^* | 1.1x10^-3^ | 0.45 | min |
| *Buffer* | 45.2 | 11.6 | min |

**Table S4.** Parameters used for calculating characteristic times with their symbols, values, and units.

| Parameter | Symbol | Value | Units | Source |
| --- | --- | --- | --- | --- |
| Biofilm thickness | $L_{b}$ | 5x10^-4^ | m | Chosen |
| *Diffusion coefficients in water* |  |  |  |  |
| Carbon dioxide | $D_{{CO}_{2}}$ | 1.9x10^-9^ | m^2^/s | (Newman and Thomas-Alyea, 2012) |
| Protons | $D_{H^{+}}$ | 9.3x10^-9^ | m^2^/s | (McCall and Douglass, 1965) |
| Buffer | $D_{Phosphate}$ | 3.6x10^-10^ | m^2^/s | (Krom and Berner, 1980) |
| *Concentrations in the bulk liquid* |  |  |  |  |
| Carbon dioxide | $C_{{CO}_{2}}^{liquid}$ | 8.35 | mol/m^3^ | From simulations |
| Protons | $C_{H^{+}}^{liquid}$ | 10^-5.8^ | mol/L | (Jourdin et al., 2019) |
| Buffer | $C_{Phosphate}^{liquid}$ | 6.4x10^-2^ | mol/L | (Jourdin et al., 2019) |
| *Reaction rates at steady state* |  |  |  |  |
| Carbon dioxide | $r_{{CO}_{2}}^{b}$ | 120 | mol/ (m^3^ h) | From simulations |
| Protons | $r_{H^{+}}^{b}$ | 85 | mol/ (m^3^ h) | From simulations |
| Buffer | $r_{Phosphate}^{b}$ | 85 | mol/ (m^3^ h) | Same as H^+^ consumed |

CO_2_ characteristic reaction and diffusion times are in the same order of magnitude, hence it is likely that CO_2_ diffusion in the biofilm limited its uptake rate. For H^+^ it is clear that the diffusion time is much larger than the time of reaction, therefore it is safe to say that protons were not able to diffuse into the biofilm fast enough. However, the presence of a buffer has to be assessed (**Figure S3**). The diffusion time of the protonated compound is of the same order of magnitude to that of CO_2_, hence it is the reaction rate that limited its deprotonation. Since the microbial reaction cannot be faster than the substrate diffusion rate, CO_2_ diffusion limitation slowed down biological rates, subsequently limiting proton consumption. This decrease in the uptake of protons might have been enough to give enough time to the buffering reaction and prevent a large pH gradient, as seen by Vander Wielen et al. (1997).


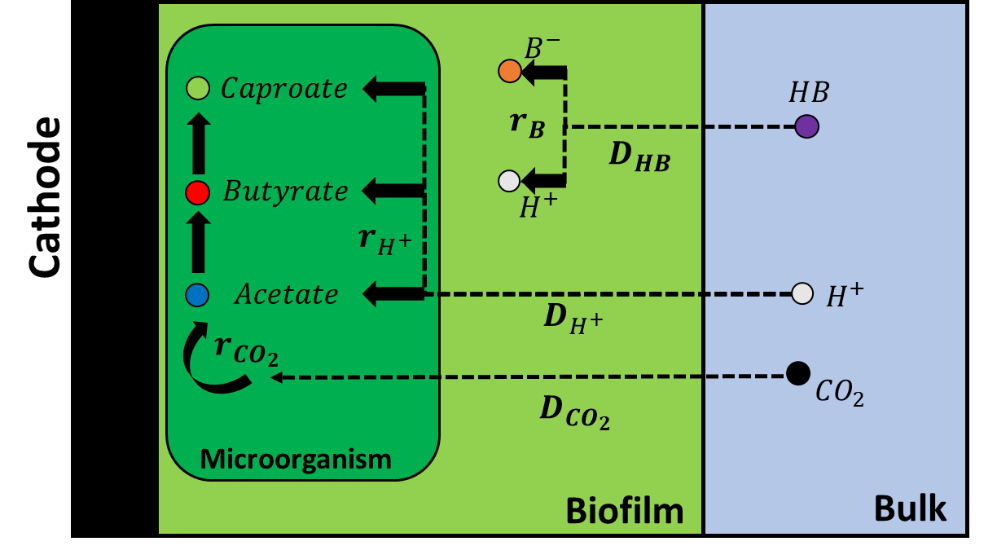


**Figure S3.** Buffering mechanism within the biofilm. Dashed arrows indicate diffusion mechanisms, solid arrows indicate reactions. B^-^ refers to the buffer compound and HB to its protonated form.

1. **References for the SI**

Bertsch, J., C. Öppinger, V. Hess, J. D. Langer and V. Müller (2015). "Heterotrimeric NADH-oxidizing methylenetetrahydrofolate reductase from the acetogenic bacterium Acetobacterium woodii." Journal of bacteriology **197**(9): 1681-1689.

Furdui, C. and S. W. Ragsdale (2000). "The role of pyruvate ferredoxin oxidoreductase in pyruvate synthesis during autotrophic growth by the Wood-Ljungdahl pathway." Journal of Biological Chemistry **275**(37): 28494-28499.

Jourdin, L., S. M. Raes, C. J. Buisman and D. P. Strik (2018). "Critical biofilm growth throughout unmodified carbon felts allows continuous bioelectrochemical chain elongation from CO2 up to caproate at high current density." Frontiers in Energy Research **6**: 7.

Jourdin, L., M. Winkelhorst, B. Rawls, C. J. N. Buisman and D. P. B. T. B. Strik (2019). "Enhanced selectivity to butyrate and caproate above acetate in continuous bioelectrochemical chain elongation from CO2: Steering with CO2 loading rate and hydraulic retention time." Bioresource Technology Reports: 100284.

Kleerebezem, R. and M. C. Van Loosdrecht (2010). "A generalized method for thermodynamic state analysis of environmental systems." Critical Reviews in Environmental Science and Technology **40**(1): 1-54.

Kracke, F., B. Virdis, P. V. Bernhardt, K. Rabaey and J. O. Krömer (2016). "Redox dependent metabolic shift in Clostridium autoethanogenum by extracellular electron supply." Biotechnology for biofuels **9**(1): 1-12.

Krom, M. D. and R. A. Berner (1980). "The diffusion coefficients of sulfate, ammonium, and phosphate ions in anoxic marine sediments 1." Limnology and Oceanography **25**(2): 327-337.

Madigan, M. T., J. M. Martinko and J. Parker (1997). Brock biology of microorganisms, Prentice hall Upper Saddle River, NJ.

Marshall, C. W., D. E. Ross, E. B. Fichot, R. S. Norman and H. D. May (2013). "Long-term operation of microbial electrosynthesis systems improves acetate production by autotrophic microbiomes." Environmental science & technology **47**(11): 6023-6029.

McCall, D. W. and D. C. Douglass (1965). "The effect of ions on the self-diffusion of water. I. Concentration dependence." The Journal of Physical Chemistry **69**(6): 2001-2011.

Newman, J. and K. E. Thomas-Alyea (2012). Electrochemical systems, John Wiley & Sons.

Schuchmann, K. and V. Müller (2014). "Autotrophy at the thermodynamic limit of life: a model for energy conservation in acetogenic bacteria." Nature Reviews Microbiology **12**(12): 809.

Spirito, C. M., H. Richter, K. Rabaey, A. J. Stams and L. T. Angenent (2014). "Chain elongation in anaerobic reactor microbiomes to recover resources from waste." Current opinion in biotechnology **27**: 115-122.

Vander Wielen, L., M. Van Buel, A. Straathof and K. C. A. Luyben (1997). "Modelling the enzymatic deacylation of penicillin G: Equilibrium and kinetic considerations." Biocatalysis and Biotransformation **15**(2): 121-146.
